# Effect of Power Plant Ash and Slag Disposal on the Environment and Population Health in Ukraine

**DOI:** 10.5696/2156-9614-11.31.210910

**Published:** 2021-08-17

**Authors:** Oleksandr Popov, Andrii Iatsyshyn, Valeriia Kovach, Volodymyr Artemchuk, Iryna Kameneva, Oksana Radchenko, Kyrylo Nikolaiev, Valentyna Stanytsina, Anna Iatsyshyn, Yevhen Romanenko

**Affiliations:** 1 Institute of Environmental Geochemistry, National Academy of Sciences of Ukraine, Kyiv, Ukraine; 2 G.E. Pukhov Institute for Modelling in Energy Engineering, National Academy of Sciences of Ukraine, Kyiv, Ukraine; 3 Interregional Academy of Personnel Management, Kyiv, Ukraine; 4 National Aviation University, Kyiv, Ukraine; 5 Institute of General Energy, National Academy of Sciences of Ukraine, Kyiv, Ukraine

**Keywords:** thermal power plants, ash dumps, ash-slag wastes, environmental contamination

## Abstract

**Background.:**

Ash and slag disposal areas of Ukrainian thermal power plants accumulate large amounts of waste annually.

**Objectives.:**

The aim of the present study was to analyze the composition of ash and slag wastes generated during combustion of coal at Ukrainian thermal power plants and the potential affects of disposal areas on the environment.

**Methods.:**

A literature search was conducted using the Google search engine to access online academic publications indexed in Google Scholar, PubMed, Scopus, Clarivate Analytics (Web of Science), ScienceDirect, ResearchGate and Springer Link from 2011 in English and Ukrainian.

**Results.:**

After analyzing the 25 academic articles included in the present review, the results indicated that hazardous constituents (oxides of silicon, aluminium, iron, calcium and magnesium) of ash can migrate from the ash dump surface by air and water to contaminate the atmosphere, soil, groundwater and surface water in areas located within a few kilometers from the waste site.

**Conclusions.:**

By-products of the fuel and energy complex of Ukraine are potentially dangerous sources of environmental pollution. They create risks to the health of the population living in the surrounding territories. Further studies should focus on the features of pollutant transfer from ash dumps, and development of appropriate mathematical models of the pollutant migration to assess pollution levels in soil, groundwater and air.

**Competing Interests.:**

The authors declare no competing financial interests.

## Introduction

**Figure 1:**
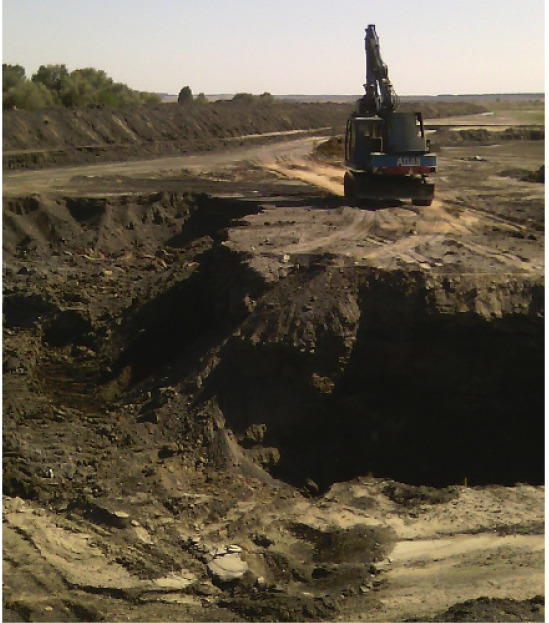
Ash and slag dump of Ladyzhynska TPP (Ladyzhyn city)

Air is heavily polluted by industrial emissions containing various harmful substances from the environment (sulfur, nitrogen, carbon, heavy metals, hydrocarbons, dust particles) in many regions of Ukraine.^1^ One of the sources of atmospheric pollution is a by-product of thermal power plants (TPP) and cogeneration plants (CP) that burn solid fuel.^1^ Consumption of electricity and heat has increased in Ukraine in recent years, leading to an increase in solid fuel combustion.^1^ It also increases the amount of solid waste stored in the landfill. Ukraine accumulates 8 million tons of ash and slag waste annually, covering an area of more than 22 thousand hectares.^1^ A one million-kilowatt power plant burns approximately 10 000 tons of coal daily with an output of 1000 tons of slag and ash (10% ash content). About one ha of land is required in order to dispose of this amount of waste to the height of the dump no more than 8 meters. For example, approximately 500 000 tons of ash and slag are formed annually at the territory of Ladyzhynska TPP (Ladyzhyn, Vinnytsia region) according to reports.^1^ Currently, the site contains 30 million tons of ash and slag mixtures at a height of 35 m over a 120-ha area. Figure 1 shows the ash dump of Ladyzhynska TPP. This ash dump is one of the most hazardous industrial waste sites in Ukraine in terms of volume and geographical location.

High income countries use ash and slag waste as a raw material for construction of skyscrapers, road construction, internal strengthening of mines, railway tracks, etc. This use saves on natural resources such as sand, reduces the cost of finished products, and reduces the level of environmental pollution. Today, about 70–95% of coal waste is recovered in the European Union (EU), and 100% in the Netherlands and Denmark. The level of waste recovery in Ukraine is only about 15%, which is extremely low compared to elsewhere in the world. Environmental damage is caused by aerosol generation from the surface of dry ash and the filtration of contaminated water through poorly shielded ash dumps from ash and slag storage sites at power plants. The aerosolization of surface of ash is a significant factor contributing to environmental degradation.

Research on the influence of energy by-products on the state of environmental safety^2^ has demonstrated the influence of the Trypil`ska TPP ash dump on the adjacent population's health. Previous work^3^ has analyzed the influence of Chinese thermal power plants on the environment. Other studies^4^–^8^ have addressed issues of pollutant distribution in the environment caused by emissions of potentially hazardous by-products. One report^9^ contained an analysis of monitoring data and assessment of groundwater status near the Sumy cogeneration plant. Other authors^10^ analyzed harmful emissions of the Burshtyn thermal power plant and surrounding environmental conditions. The development of monitoring is described in a number of studies.^11^–^16^ Issues related to the mathematical and software tools, and the economic impact of the by-products on the environment are highlighted.^16^–^21^ Other documents describing solutions for processing of ash and slag were reviewed. These include the Asian Coal Ash Association,^22^ the European Coal Combination Products Association,^23^ and the American Coal Ash Association.^24^

**Abbreviations**
*CP*: Cogeneration plant
*MPC*: Maximum permissible concentration
*TPP*: Thermal power plant

These reports do not sufficiently cover the analysis of full-scale (laboratory) observations of harmful substances contained in the ash wastes accumulated in the landfills of various solid fuel power plants in Ukraine. Furthermore, they failed to assess the environmental impact of these substances. The review indicates a need for research in this area. Expanded knowledge makes it possible to determine directions and prospects for ash/slag reuse with knowledge of waste chemical composition and technical properties in order to reduce environmental and human health impacts. The present study was conducted in order to analyze the composition of ash-slag wastes generated during the burning of coal at TPPs in Ukraine and the impact of waste storage sites on the environment.

## Methods

**Figure 2:**
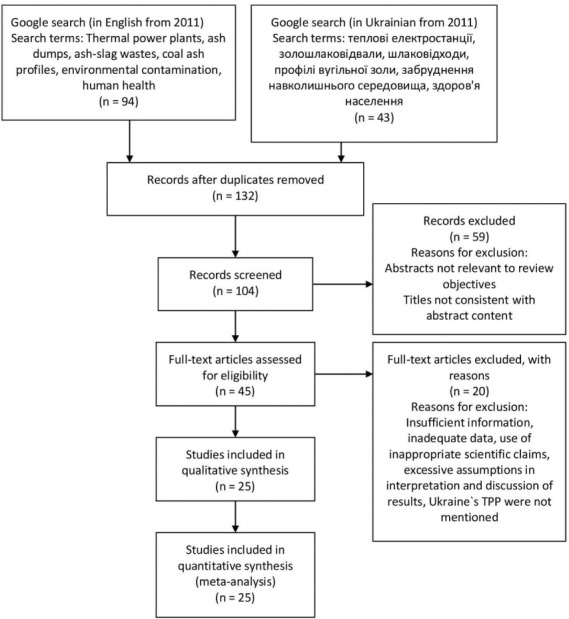
PRISMA^25^ flow chart showing literature collection, screening, inclusion, and exclusion procedure

The present study is a literature review addressing the impact of ash and slag waste on the environment. Assessments included comparative analysis of the impact of ash-slag waste monitoring methods and the impact of ash-slag waste disposal areas on the environment and the population. A systematic literature search was conducted using the Google search engine to access online academic publications indexed in Google Scholar, PubMed, Scopus, Clarivate Analytics (Web of Science), ScienceDirect, ResearchGate and Springer Link from 2011 in English and Ukrainian. The words, terms, clauses, and phrases used in the literature search were ‘thermal power plants', ‘ash dumps', ‘ash-slag wastes,' ‘coal ash profiles', ‘environmental contamination', and ‘population health' in English, and ‘теплові електростанції’, ‘золошлаковідвали’, ‘шлаковідходи’, ‘профілі вугільної золи’, ‘забруднення навколишнього середовища' and ‘здоров'я населення' in Ukrainian. A total of 132 peer reviewed publications were accessed based on the relevance of titles to the study. These were further screened to 104 after reading through their abstracts. After screening the full length of the papers, 25 were used for this review. Articles were excluded as a result of irrelevance of abstracts to our review objectives, inconsistencies of titles with abstract and full-length content, inadequate data presentation, use of inappropriate statistical tools, unsubstantiated scientific claims, or excessive assumptions in interpretation and discussion of results. The last day of article search was April 7, 2021. Literature collection, screening, inclusion, and exclusion procedures are shown in Figure 2.

## Results

Table 1 summarizes the results of by study focus areas: ash disposal areas of TPPs, composition of ash and sludge wastes, the qualitative chemical composition of ash wastes, and content of radionuclides in the ash and slag of TPP.

**Table 1:** Included Studies by Focus Area

**Focus area**	**Reference**
Ash disposal areas of TPPs	Shkitsa et al., 2013^8^
Composition of ash and sludge wastes	O.M. Marzieiev Institute for Public Health, 2016^2^, Kryzhanivsky et al., 2016^10^, Delitzin et al., 2012^24^
The qualitative chemical composition of ash wastes	O.M. Marzieiev Institute for Public Health, 2016^2^, Kryzhanivsky et al., 2016^10^, Clarke et al., 1924^21^, Rentier et al, 2019^23^, Delitzin et al., 2012^24^
Content of radionuclides in the ash and slag of TPP	Popov et al., 2019^17^, Pribilova, 2013^25^, Hobotova et al., 2012^26^

Ash disposal areas of TPPs are special hydrotechnical facilities designed for storage of ash wastes. They are contained by dams and terrain. As a rule, hydro removal systems are usually used for transport of water from the territory of power plants to the ash dump. In such systems, water-mixed slag ash in the form of a pulp enters the ash dump through the pipeline. The pulp is divided into clarified water and sedimentation of solid particles. There are two processes in the ash dump. The first is evaporation of water and the formation of so-called “beaches” in the ash dump. The “beach” is a dry area of dust particles. The second process is water infiltration (clarified or even partially untreated) and ingress of dissolved forms of toxic components of ash-slag into the groundwater and reservoirs outside the ash dump.

**Figure 3:**
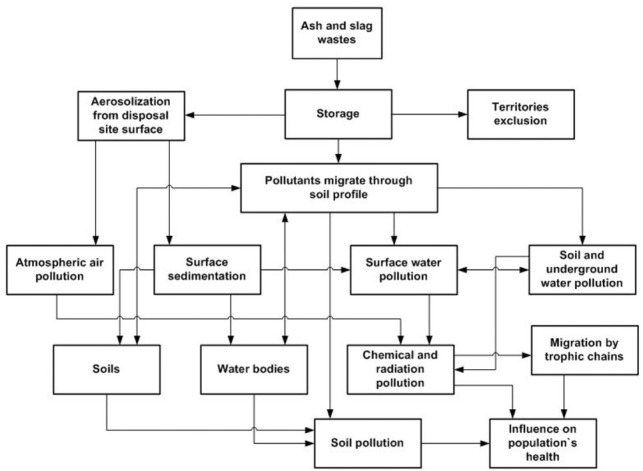
Scheme of influence of ash and slag disposal sites on the environment

Thus, ash and slag disposal areas of power plants are open systems within the fenced area. Hazardous constituents of ash can migrate from the ash dump surface through the air and water action contaminate the atmosphere, soil, groundwater and surface water in areas located a few kilometers from the waste accumulator. Toxic substances contained in the ash waste can cause degradation of the biosystem (flora and fauna, hydrobionts) and adversely affect human health (through water, air, food) spreading across the different trophic chains. Figure 3 shows the interaction scheme of the ash disposal area with the environment.^9^

### Composition of ash and sludge wastes

It is important to examine the mineralogical, elemental, oxide and radionuclide composition of the waste, particle surface structure of the particles, sorption and hydraulic activity, and behavior of minerals during heating, in order to determine the precise properties of ash-slag waste.

Slag and ash are quite toxic, and their toxicity consists of heavy metals and unidentified organic toxicants. In chemical composition, ash pollutants are a complex mixture of primarily mineral substances. Composition of various chemical compounds in the ash waste depends on fuel composition. By-products of the fuel and energy complex of Ukraine are produced from coal fuel of different brands. Therefore, concentrations of ash components vary within certain limits, but in all cases the main ash-forming components are oxygen compounds of silicon, aluminum, iron, calcium and magnesium. A small proportion of elements is present in the ash wastes in the form of CaSO_4_, MgSO_4_ and FeSO_4_ sulfates. Table 2 shows average chemical composition of the mineral part of ash wastes from solid fuel of TPPs in Ukraine and Russia.^2^,^10^,^27^ One report^27^ found the bulk (96–98%) of ash waste to contain on average: silicon oxide, 45–60%; calcium oxide, 2.5–9.6%; magnesium oxide, 0.5–4.8%; iron oxide, 4.1–10.6%; aluminum oxide, 10.1–21.8%; and sulfur trioxide, 0.03–2.7%.

**Table 2:** Average Chemical Composition Profiles of the Mineral Content of Ash Wastes from Solid Fuel at Thermal Power Plants and Cogeneration Plants in Ukraine and Russia^2^,^27^

**Enterprise title**	**Mineral ash and slug formation components, mass. %**							
	SiO_2_	Al_2_O_3_	Fe_2_O_3_	CaO	MgO	Na_2_O	K_2_O	SO_3_
Starobeshivska TPP	44–50	24–30	8–6	2.5–4.6		-		1.2
Kramatorska CP	49.4	22.5	17.1	10.05		-		
Vuhlehirska TPP	16.7	40.6	23.0	8.4		-		0.02
Myronivska TPP	67.8	19.8	10.9	5.5		2.8		2.1–7
Burshtynska TPP	48.6	23.3	15.3	4.4	2.2	0.5	1.6	0.47
Vladyvostotska CP-2 (Russia)	10–58	10–30	2–20	2–60	0–10	0–5	0–5	-
Krasnoiarska CP-1 (Russia)	48.6	8.0	10.3	29.7	5.7	0.3	0.3	1.5
Clarke content in the earth’s crust [22]	55.3	14.3	6.7	4.8	3.3	3.2	3.1	-

Abbreviations: CP, cogeneration plant; TTP, thermal power plant

The qualitative chemical composition of ash wastes is close to sedimentary rocks and at first glance ash dumps do not pose an environmental hazard. However, ash damps are a hazard. The constituents in ash waste can significantly exceed the value of their concentrations in the earth's crust.^21^

Thus, CaO composition in ash waste may be higher than the Clarke value by 4–12 times, Al_2_O_3_ by 2 times, Fe_2_O_3_ by 1.5–3 times, and MgO by 2–3 times. Storage of such wastes in natural conditions can lead to disturbance of the ecological balance. In addition, ash slag is very different from sedimentary rocks in phase-mineralogical composition. They consist of amorphous-glassy substances (formed by silicates, aluminosilicates, ferroalumosilicates, a number of free oxides, mainly CaO) and number of crystalline hematulates, magnesites and other minerals. Among the components of both mineral phases are substances that pose real threat to human and animal health (especially when they are released into the air). Glass-silicate dust adversely affects the pulmonary-respiratory system, calcium oxide in the form of dust irritates the mucous membranes. Water becomes aggressive when the dust enters it and changes the pH.^26^

The most hazardous crystals are quartz and mullite, which cause fibrotic changes in the lung tissue. Thus, inorganic dust containing quartz is classified as hazard class III by sanitary standards; the value of daily maximum permissible concentration (MPC) for it in the atmospheric air is 0.05–0.15 mg/m^3^ (depending on the content of SiO_2_ in the dust). Almost all hazardous mineral macro-constituents of ash waste (silicates, quartz, mullite) are poorly released from the human body and their negative impact over time (with prolonged contact with ash-slag dust) is amplified.

In addition to the listed number of harmful macro-constituents of ash waste there are also large number of trace elements related to I-III hazard classes. However, concentrations of individual toxic trace elements in the soil can substantially exceed their Clarke value in the earth's crust and exceed the level of MPC set for them by sanitary standards. These impurity elements are called potentially toxic trace elements. Their spectrum is mainly represented by heavy metals, among which are radioactive elements (thorium, uranium, etc.). Most often, such potentially toxic trace elements (lead, copper, zinc, cadmium, chromium, nickel, etc.) are present in the coal-fired TPP of Ukraine. Table 3 shows some toxic trace elements in composition of ash-slag wastes generated by combustion of coal at some TPPs of Ukraine.^2^,^10^,^27^ The composition of these hazardous components in ash slag can exceed the level of MPC (in soil). As these trace elements enter the solid fuel combustion concentrate, their content in the ash slag is directly determined by the brand of coal used by the power plant. Different coal sources have different chemical profiles.

**Table 3:** Toxic Trace Elements in the Composition of Ash-slag Waste Generated by the Burning of Coal at Thermal Power Plants in Ukraine 2,10,27

**Chemical element**	**MPC in soil, mg/kg**	**Clarke, mg/kg**	**Content in ash and slag wastes, mg/kg**				**Class of danger/ Toxic action**

			Trypilska TPP	Darnytska CP	Cherkaska CP	Burshtynsk a TPP	
Zinc (Zn)	100.0	50	229.0	70.4	18.02	36.6	I class. Toxic heavy metal with high volatility
Cadmium (Cd)	N/A	0.5	0.2	0.2	N/A	0.9	I class. Toxic heavy metal, affects respiratory system and internal organs (liver, kidneys)
Lead (Pb)	32.0	10	6.7	2.1	0.28	23.1	I class. Acts on the nervous system
Copper (Cu)	55.0	20	28.6	22.7	45.44	19.8	II class. Causes diseases of respiratory system, nervous system, gastrointestinal tract and liver
Nickel (Ni)	85	40	5.1	54.41	2.32	27.3	II class. Affects respiratory organs, has carcinogenic and allergenic properties
Chromium (Cr)	N/A	200	40.9	30.7	0.54	N/A	II class. Affects respiratory system, cardiovascular system and internal organs (liver, kidneys)
Manganese (Mn)	1500	850	N/A	106.1	N/A	27.3	III class
Iron (Fe)	N/A	38000	1014,65	N/A	N/A	N/A	III class
Aluminium (Al)	N/A	71300	1088.61	N/A	N/A	N/A	III class

Abbreviations: CP, cogeneration plant; MPC, maximum permissible concentration; TTP, thermal power plant

N/A = data are not available

Content of radionuclides in the ash and slag of TPP (CP) also should be determined. This indicator depends on the characteristics of the coal and the technology of combustion. It is known that technology of coal combustion in a circulating fluidized bed (CCS) can significantly improve economic and environmental performance of the heaters. Table 4 shows the content of natural radionuclides in the ash-slag mixture of thermal power plants, which is formed by different methods of energy combustion.^2^ The table shows that the concentration of radionuclides in the ash of the boiler plants is much higher than in other coal combustion technologies. Studies on the chemical composition of the Zmiivska TPP showed the presence of 40K, 226Ra, 232Th, 137Cs, but level of ash radioactivity was 4–5 times lower than the maximum permissible level for soil.^28^ Other chemical elements are also often found in ash waste: strontium (Sr), Cr, gallium (Ga), beryllium (Be), cobalt (Co), bismuth (Bi), sliver (Ag), and arsenic (As).^28^,^29^

**Table 4:** Content of Natural Radionuclides in the Ash-slag Mixture of Thermal Power Plants Formed by Different Methods of Energy Combustion

	**Specific activity of radionuclides, Bq/kg**			**Total specific activity, Bq/kg**
	Th-232	Ra-226	K-40	
Ash	55±11	101±20	646±129	228±46
Ash-sludge	28±6	65±13	468±94	142±28

## Discussion

Toxic trace elements in ash can contaminate soil, atmospheric air and water. They can get into the air due to the high dispersion of ash and slag dust. Trace elements in mobile (water-soluble) forms can pass into water. Levels of MPCs of toxic trace elements in atmospheric air and water are more stringent than the values of MPC in soil. Air and water pollution pose the greatest environmental risk, as this pollution leads to rapid involvement of toxic trace elements in natural exchange processes. Pollution of reservoirs is particularly hazardous, as toxins can bioaccumulate and biomagnate. For example, movement through the trophic chain: water → algae → invertebrates → fish → animals → humans. The content of hazardous trace elements in algae, invertebrates and fish can exceed their concentration in the source water by 10–100 times, i.e. when they enter the biosphere toxic components are accumulated and their action is enhanced.^27^ Ash wastes contain mineral micro- and macro-constituents, organic substances that are associated with partial combuston of carbon. However, if combustion is disturbed (and this often happens in practice), content can increase significantly.

Hazardous constituents of ash can migrate from the surface of the ash dump through the air and water environment and pollute surface layer of the atmosphere, soil, groundwater and surface water in areas located few kilometers away from the waste accumulator. Studies showed that chemical composition of ash waste is a complex mixture of different substances, and their content depends on composition of fuel.^27^ By-products of the fuel and energy complex of Ukraine are produced from coal fuel of different brands. The concentrations of components in ash-slag waste vary within certain limits, but in all cases the main ash-forming components are oxygen compounds of silicon, aluminum, magnesium, iron.^27^

The content of components in ash slag can significantly exceed the value of their concentrations in the earth's crust: CaO may be higher than the Сlark value by 4–12 times, Al_2_O_3_ by two-fold, Fe_2_O_3_ by 1.5–3 times, and MgO by 2–3 times. Often lead, copper, zinc, cadmium, chromium, nickel, etc., as well as radionuclides are present in the coal wastes of Ukraine.^27^

Toxic and mineral components are present in the mineral phase and in organic particulates. They pose the greatest danger when exposed to air in the composition of dust (quartz, mullite, toxic elements—impurities, condensed aromatic hydrocarbons) and/or in water (calcium oxide, toxic trace elements in mobile, water-soluble form). However, harmful substances that are in the composition of ash waste can have a cumulative effect. They can lead to the degradation of the ecosystem and the emergence of various human diseases, including cancer.^27^

## Conclusions

By-products of Ukraine's fuel and energy complex are potentially dangerous sources of environment pollution, and they create health risks for the population living in the surrounding area. Further studies should be focused on features of pollutant transfer in the areas of ash dumps, and on the construction of appropriate mathematical models for migration of pollutants to assess levels of contamination in soil, groundwater and in the air. The authors emphasized the issues of ecological safety and the recycling prospects of ash and slag materials. The present study is aimed at monitoring and evaluating the environmental impact of ash-slag. It should attract the attention of specialists addressing the problem of ash-slag waste utilization in Ukraine, while taking into account global experiences and reducing harmful effects of this waste in the future.
